# Diagnostic performance of T1- and T2-mapping in HTx patients to identify acute cellular rejection (ACR) in comparison to conventional CMR techniques and endomyocardial biopsy (EMB) as the standard of reference

**DOI:** 10.1186/1532-429X-17-S1-Q54

**Published:** 2015-02-03

**Authors:** Lysann Reinhardt, Matthias Gutberlet

**Affiliations:** 1 Department of Diagnostic and Interventional Radiology, University Leipzig - Heart Center Leipzig, Leipzig, Germany

## Background

Cardiovascular magnetic resonance (CMR) is an excellent non invasive method for lifelong post HTX staging, feasible to identify patients with an acute cellular rejection (ACR). This study evaluates the diagnostic performance of T1- and T2-Mapping CMR to identify biopsy proven acute cellular rejection (ACR) in comparison to conventional CMR techniques.

## Methods

Thirty-five CMRs were performed (mean age 53±11 years, 24male) using a 1.5T scanner (Achieva, Philips medical systems, Best, The Netherlands) compared to EMB. The CMR-protocol included conventional sequences to assess myocardial edema ratio (ER), a T1-weighted spinecho sequence for global relative enhancement (gRE) and inversion recovery sequences to visualize late gadolinium enhancement (LE). Histological grading according to the International Society for Heart and Lung Transplantation (ISHLT) from 1990, in which grade ≥1B was considered as a clinically relevant ACR, which has to be treated.

T1-quantification was performed using the modified Look-Locker inversion-recovery (MOLLI) sequence before and 15 minutes after administration of 0.1 mmol/kg body weight of Gadobutrol (Gadovist, Bayer HealthCare, Berlin, Germany). T2-quantification was performed using a free-breathing, navigator-gated multi-echo-sequence. Global myocardial T1 pre- and post-contrast, T2 and ECV maps were calculated with a dedicated Software (cvi42).

## Results

In 20/35 (57%) EMBs there was no or mild ACR (ISHLT 0A, 1A) and a clinically relevant ACR in 15/35 EMBs (1B, 2A, 4A).

The best Receiver Operating Characteristics (ROC) with high values for the area-under-the-curve (AUC) - to discriminate between relevant and non-relevant ACR - were demonstrated by postcontrast myocardial T1-mapping (0.78), myocardial T2-mapping (0.73) and native myocardial T1-mapping (0.65), respectively. Comparable results were achieved with the calculated value of the extracellular volume (ECV) with 0.66. The conventional CMR techniques ER and gRE revealed only moderate results with 0.54 and 0.52, respectively. Similar to myocarditis the critical values for ER were ≥2 and ≥4.5 for the gRE.

Accordingly, the best sensitivity and specificity for clinically relevant ACR ≥1B could be achieved with postcontrast T1-mapping using a cut off-value of 342ms (73%/70%) and T2-mapping using a cut off value of 65ms (73%/75%), respectively. Again comparable results could be achieved with the ECV using a cut off of 42 (67%/70%) and moderate results by using native T1-mapping with a cut off of 1060 ms (87%/45%).

## Conclusions

Parametric CMR is encouraging to identify subclinical ACR in patients after HTx, especially postcontrast T1- and T2-mapping. However, larger studies are needed to underline this and potentially reduce, or eliminate the need for EMB.

## Funding

In-house research funds.

